# Antilipotoxicity Activity of *Osmanthus fragrans* and *Chrysanthemum morifolium* Flower Extracts in Hepatocytes and Renal Glomerular Mesangial Cells

**DOI:** 10.1155/2017/4856095

**Published:** 2017-11-20

**Authors:** Po-Jung Tsai, Mei-Ling Chang, Ching-Mei Hsin, Chung-Chieh Chuang, Lu-Te Chuang, Wen-Huey Wu

**Affiliations:** ^1^Department of Human Development and Family Studies, National Taiwan Normal University, Taipei, Taiwan; ^2^Department of Food Science, Nutrition and Nutraceutical Biotechnology, Shih Chien University, Taipei, Taiwan; ^3^Department of Biotechnology and Pharmaceutical Technology, Yuanpei University of Medical Technology, Hsinchu, Taiwan

## Abstract

The excess influx of free fatty acids (FFAs) into nonadipose tissues, such as those of liver and kidney, induces lipotoxicity leading to hepatic steatosis and renal dysfunction. The aim of this study was to investigate the protective effects of methanolic flower extracts of *Osmanthus fragrans* (OF) and *Chrysanthemum morifolium* (CM) against FFA-induced lipotoxicity in hepatocytes (human HepG2 cells) and renal glomerular mesangial cells (mouse SV40-Mes13 cells). The results showed that OF and CM significantly suppressed FFA-induced intracellular triacylglycerol accumulation via partially inhibiting the gene expression of sterol regulatory element-binding protein-1c (SREBP-1c) and glycerol-3-phosphate acyltransferase (GPAT) in HepG2 cells. Both extracts inhibited reactive oxygen species (ROS) generation by FFA-stimulated HepG2 cells. OF and CM also suppressed the mRNA expression of interleukin- (IL-) 1*β*, IL-6, IL-8, tumor necrosis factor- (TNF-) *α*, and transforming growth factor- (TGF-) *β* by HepG2 cells treated with conditioned medium derived from lipopolysaccharide-treated THP-1 monocytes. Furthermore, OF and CM effectively inhibited oleate-induced cellular lipid accumulation, TGF-*β* secretion, and overexpression of fibronectin in mesangial cells. In conclusion, OF and CM possess hepatoprotective activity by inhibiting hepatic fat load and inflammation and renal protection by preventing FFA-induced mesangial extracellular matrix formation.

## 1. Introduction

Lipotoxicity is generally defined as an increased concentration of harmful lipids, leading to cellular dysfunction and disruption of tissue function. The different classes of free fatty acids are known to trigger toxic effects and inflammation in numerous cell types [1]. Lipotoxicity may occur in several target organs via direct effects of triggering inflammation pathways and through indirect effects of alterations in the gut microbiota associated with endotoxemia [2]. Lipotoxicity plays a critical role in the pathogenesis of nonalcoholic fatty liver disease (NAFLD) and renal diseases [3, 4]. Nonalcoholic steatohepatitis (NASH), an advanced form of NAFLD that may progress to cirrhosis, is caused by lipid-mediated toxicity and inflammatory responses [3]. Intracellular lipid accumulation in NAFLD results from increased fatty acid uptake, increased de novo lipogenesis, and decreased fatty acid oxidation followed by esterification for the triacylglycerol (TG) synthesis. Lipogenesis is controlled primarily at the transcriptional level. Sterol regulatory element-binding protein 1c (SREBP-1c) and carbohydrate response element-binding protein (ChREBP) have been described as major transcription factors for increased de novo lipogenesis in NAFLD [5]. Glycerol-3-phosphate acyltransferase (GPAT) which catalyzes the esterification of glycerol-3-phosphate with fatty acid to generate lysophosphatidic acids is the rate-limiting enzymes of TG synthesis, and its gene expression is activated by SREBP-1c [5].

High level of plasma lipids may contribute to renal lipid accumulation, generation of reactive oxygen species (ROS), mesangial expansion, and development of glomerulosclerosis [4]. Many studies indicate that transforming growth factor *β* (TGF-*β*) plays a central role in the pathogenesis of renal fibrosis and is an important marker of early renal fibrosis. TGF-*β* exerts profibrotic activity through stimulation of fibroblast proliferation and epithelial-mesenchymal transition (EMT). The increased TGF-*β* stimulates glomerular ECM accumulation by stimulating mesangial cells to produce type I, III, and IV collagen; laminin; and fibronectin and by blocking matrix degradation [6].

Flowers of *Osmanthus fragrans* and *Chrysanthemum morifolium* are commonly used as folk medicine and additives for teas, beverages, and foods in Taiwan. *O. fragrans* flowers (also known as Kwai-fah in Chinese) have been used to relieve pain, coughing, stomachache, diarrhea, and hepatitis in traditional Chinese medicine (TCM). Various compounds have been isolated from *O. fragrans* flowers, including flavonoids, phenolic acids, tyrosyl acetate, phillygenin, ligustroside, and verbascoside [7–9]. *O. fragrans* flower extract and its bioactive components have shown anti-inflammatory, antioxidant, and neuroprotection activity, alleviating diabetic pathological conditions and attenuating acetaminophen-induced hepatotoxicity [9–11]. *Chrysanthemum morifolium* flowers (also known as Ju-hua in Chinese) have been used in TCM as a medication for common cold, dim eyesight, dizziness, and skin itch. This flower is also widely used as a food supplement, or herbal tea, and is considered a healthy food by many consumers [12]. There are several cultivars of *C. morifolium* flowers available in herb markets in Taiwan; “Taiwan Hang Ju” is often used as herbal tea or beverage. *C. morifolium* flowers contain many phenolic compounds such as flavonoids, caffeic acid derivatives, hydroxycinnamoylquinic acids, and triterpenoid compounds [12]. Our previous study showed that selective phenolics, including chlorogenic acid, quercetin, myricetin, and caffeic acid, were identified in the methanol extracts of *C. morifolium* and *O. fragrans* flowers [7]. *C. morifolium* flower extract and its components also possess a variety of biological characteristics such as antioxidant, anti-inflammatory, antivirus, anti-HIV, antimutagenic, anticarcinogenic, and antiaging activities [12, 13]. In addition, polyphenol-rich *C. morifolium* extract ameliorated high-fat/drug-induced fatty liver in mice by orally feeding emulsion containing 10% cholesterol, 20% lard, and 0.2% propylthiouracil [14]. Propylthiouracil is known to cause liver injury and acute liver failure [15].

Despite numerous known biological functions of flower extracts of *O. fragrans* and *C. morifolium*, limited information is available on their effects on FFA-induced hepatic and renal lipotoxicity. In this study, we examined the effect of extracts of *O. fragrans* and *C. morifolium* flowers on lipotoxicity in FFA-overloaded hepatocytes (human HepG2 cells) and renal glomerular mesangial cells (mouse SV40-Mes13 cells).

## 2. Materials and Methods

### 2.1. Materials

HepG2 cells (BCRC RM60025; human hepatoblastoma cell line), THP-1 cells (BCRC 60430; human monocytic cell line), and mesangial SV40-Mes13 cells (BCRC 60366; mouse glomerular mesangial cell line) were obtained from the Bioresource Collection and Research Center, Hsinchu, Taiwan. HepG2 cells were cultured in DMEM/high glucose (Gibco, Carlsbad, CA, USA) supplemented with 10% heat-inactivated fetal bovine serum (FBS, Gibco), 1% nonessential amino acid (Gibco), 1% L-glutamin (Gibco), and 1% penicillin/streptomycin (Gibco). Monocytic THP-1 cells were maintained in RPMI 1640 (Gibco) supplemented with 10% heat-inactivated FBS (Gibco) and 1% penicillin/streptomycin (Gibco). Mesangial cells were cultured in DMEM/low glucose/F12 medium (Gibco) supplemented with 5% FBS (Gibco) and 1% penicillin/streptomycin (Gibco). These cell lines were at 37°C in a humidified atmosphere with 5% CO_2_.

BSA (Sigma-Aldrich, St. Louis, MO, USA) solutions (10%) were prepared using phosphate-buffered saline (PBS, pH 7.2). All free fatty acids (FFAs) were purchased from Sigma-Aldrich. FFA-bovine serum albumin (FFA/BSA) complex solution was prepared as reported previously [16]. The FFA/BSA or oleic acid (OA)/BSA complex solution was sterile-filtered through 0.22 *μ*m sterile filters (Millipore S.A.S., Molsheim, France) and then stored at −20°C until use.

### 2.2. Preparation of Flower Extracts

Flowers of *Osmanthus fragrans* and *Chrysanthemum morifolium* (Taiwan Hang Ju) were, respectively, collected from Shiding, New Taipei, and Tongluo, Miaoli, Taiwan. The voucher specimens were deposited in the Department of Human Development and Family Studies, National Taiwan Normal University. The voucher specimen of the plant was authenticated by Dr. Po-Jung Tsai. The air-dried flowers were milled into powder and extracted twice with ten volumes of methanol. The filtration was performed in an evaporated and concentrated manner under vacuum at 45°C to obtain the methanolic flower extract of *O. fragrans* (OF) and the methanolic flower extract of *C. morifolium* (CM). The yields of OF and CM were, respectively, 25 and 24% (based on the weight of dried and ground plant materials). OF and CM then were redissolved in dimethyl sulfoxide (DMSO; RDH Chemical Co., Spring Valley, CA, USA) to 200 mg/mL of stock solution for the sequential experiments.

### 2.3. Effects of OF and CM on Lipid Deposition and Inflammatory Responses in HepG2 Cells

#### 2.3.1. FFA-Treated HepG2 Cells and Determination of Cellular Viability

To mimic lipid exposures during in vivo high-fat-diet condition, FFA mixtures were used to induce lipotoxicity in HepG2 cells as previously reported by Lin et al. [17]. Stock solutions of 50 mM FFA mixtures consisting of palmitic acid, oleic acid, linolic acid, linoleic acid, and arachidonic acid (in proportions of 40 : 25 : 15 : 15 : 5) were prepared in culture medium containing 1% BSA [16, 17]. HepG2 cells (5 × 10^4^ cells/well) were cultured in 96-well culture plates and 24 h later treated with various concentrations of OF or CM for the indicated times. Cell viability was determined using the 3-(4,5-dimethylthiazol-2-yl)-2,5-diphenyl tetrazolium bromide (MTT) assay. MTT solution (Sigma-Aldrich; 100 *μ*L, 0.5 mg/mL) was added to each well and incubated at 37°C for 3 h. The reaction was terminated by replacing the MTT-containing medium with 500 *μ*L of DMSO, and the formazan salts were dissolved by gentle shaking for approximately 5 min at room temperature. The optical density (OD) of each well was measured at 550 nm using a microplate reader (Tecan, Männedorf, Switzerland). Each assay was completed in triplicate wells, and each experiment was repeated three times.

#### 2.3.2. Determination of Intracellular Lipid Content in HepG2 Cells

HepG2 cells (5 × 10^4^ cells/well) were cultured in 96-well culture plates for 24 h. Cells were then incubated for another 24 h with the indicated concentrations of OF and CM or 0.1% DMSO (as vehicle cells) in the presence of 1 mM FFAs/BSA. Control cells were incubated with 1% BSA alone. The total intracellular lipid content was evaluated by Oil Red O staining. Briefly, the cells were fixed in 4% paraformaldehyde in PBS for 1 h, stained with Oil Red O (Sigma-Aldrich) for 1 h at room temperature, and then rinsed with ddH_2_O few times to remove the excess stain. After washing and drying completely, 100 *μ*L of isopropanol was added to each well and the mixtures were incubated for 10 min, followed by gentle vibration to release Oil Red O. The extraction solution was then transferred to another 96-well plate for the measurement of OD at 510 nm by a microplate reader (BioTek, Nevada, USA).

#### 2.3.3. Determination of Cellular Cholesterol and Triacylglycerol Contents

HepG2 cells (1 × 10^6^ cells/well) were cultured in 6-well culture plates and 24 h later treated with 1 mM FFAs/BSA alone (as vehicle cells) or in combination with various concentrations of OF or CM for 24 h. Cells from each well were harvested by addition of lysis buffer, and cell proteins were assessed by the Lowry protein assay (Bio-Rad, Hercules, CA, USA). The total cholesterol (TC) and total triacylglycerol (TG) contents of whole cell lysates were measured with the colorimetric assay kit (Randox, Crumlin, Antrim, UK). Their masses in HepG2 cells were calculated and normalized to total cellular protein content.

#### 2.3.4. Effect of OF and CM on mRNA Levels of SREBP-1c and GPAT in FFA/BSA-Treated HepG2 Cells

HepG2 cells (1 × 10^6^ cells/well) were cultured in 6-well culture plates and 24 h later treated with FFAs/BSA alone (as vehicle cells) or in combination with various concentrations of OF and CM. After 24 h incubation, HepG2 cells were collected and total RNA was extracted from cells using the TRizol reagent (Invitrogen, Carlsbad, CA), and complementary DNA (cDNA) was generated from 2 *μ*g of total RNA, with the oligo (dT) primer and 1 *μ*L of reverse transcriptase (Promega, Madison, WI, USA). Real-time PCRs were conducted in an iCycler iQ Real-Time detection system (Bio-Rad, Hercules, CA, USA) using iQ™ SYBR Green Supermix (Bio-Rad). The relative amounts of the PCR products were analyzed by iQ5 optical system software, vers. 2.1. As shown in Table 1, primer sequences for SREBP-1c and GPAT were used in this study. The messenger RNA (mRNA) level of each sample for each gene was normalized to that of the glyceraldehyde-3-phosphate dehydrogenase (GAPDH) mRNA. Fold expression was defined as the fold increase relative to control cells.

#### 2.3.5. Measurement of ROS Production

The probe 2,7-dichlorofluorescein diacetate (H_2_DCF-DA; Sigma-Aldrich) was used to monitor the intracellular ROS generation. HepG2 cells (5 × 10^4^ cells/well) were seeded in a 96-well plate in DMEM medium for 24 h. Cells were then incubated for another 24 h with the indicated concentrations of OF or CM or 0.1% DMSO (as vehicle cells) in the presence of 1 mM FFAs/BSA. Control cells were incubated with 1% BSA in the absence of 1 mM FFAs/BSA. After incubation, cells were washed with PBS and incubated with 10 *μ*M H_2_DCF-DA at 37°C for 2 h. The formation of the oxidized fluorescent derivative dichlorofluorescein (DCF) was monitored at 475 nm excitation and 525 nm emission using a Synergy™ HT Multi-Mode Microplate Reader (BioTek, Nevada, USA). All procedures were performed in the dark.

#### 2.3.6. Determination of Proinflammatory Cytokine mRNA Levels in Conditioned Medium-Treated HepG2 Cells

For the preparation of conditioned medium, THP-1 cells were treated with 0.5 *μ*g/mL lipopolysaccharide (LPS) (Sigma-Aldrich) for 24 h; the medium was collected and centrifuged to remove cell debris. Culture supernatants derived from THP-1 monocytes were referred to as THP-1/LPS/conditioned media (TLPS/CM) and stored at −20°C until use. HepG2 cells were initially maintained in DMEM with 10% FBS. The RPMI 1640 medium was then gradually increased to replace DMEM, and the cells were routinely passaged when confluence was achieved. Afterwards, HepG2 cells were grown in RPMI 1640 medium supplemented with 10% FBS for the subsequent experiments. HepG2 cells (1 × 10^6^ cells/well) were seeded in a 6 cm dish with serum-free RPMI 1640 medium. HepG2 cells were cultured in RPMI 1640 medium (as control cells) and 50% TLPS/CM alone (as vehicle cells) and coincubated with 400 *μ*g/mL of OF or CM in the presence of 50% TLPS/CM. After 48 h incubation, HepG2 cells were collected and total RNA was extracted from cells using the TRizol reagent (Invitrogen, Carlsbad, CA), and complementary DNA (cDNA) was generated from 2 *μ*g of total RNA, with the oligo (dT) primer and 1 *μ*L of reverse transcriptase (Promega, Madison, WI, USA). Primer sequences were used in this study (Table 1). Real-time PCR analyses were conducted as described above.

### 2.4. Effects of OF and CM on Oleic Acid-Induced Lipotoxicity in Renal Glomerular Mesangial Cells

#### 2.4.1. OA-Treated Mesangial SV40-Mes13 Cells and Determination of Cellular Viability

Mishra and Simonson [18] found that treatment of 200 *μ*M oleate/BSA induced a myofibroblast-like phenotype in mesangial cells, which is an implication for renal fibrosis. So, we used the same methodology to examine the possible nephroprotective properties of OF and CM. Stock solutions of 50 mM OA/BSA were also prepared as previously described [16].

Mesangial SV40-Mes13 cells (1 × 10^4^ cells/well) were seeded in a 96-well plate for 24 hours and then growth-arrested for another 24 h in FBS-free medium. Mesangial cells were then incubated in DMEM/F12 medium containing 200 *μ*M OA/BSA and coincubated with various concentrations of OF or CM (50, 100, and 200 *μ*g/mL) for another 48 h. Vehicle cells were incubated with 0.1% DMSO in the presence of OA/BSA. Control cells were incubated with 1% BSA alone. Cell viability was determined by the Alamar blue assay (Invitrogen, Carlsbad, CA, USA) according to the manufacturer's protocol.

#### 2.4.2. Determination of Intracellular Lipid Content in Mesangial Cells

Mesangial SV40-Mes13 cells (3 × 10^5^ cells/well) were seeded in 6-well plates for 24 h and then growth-arrested for another 24 h in FBS-free medium. Cells were treated with OA/BSA (200 *μ*M) alone or in combination with different concentrations of OF or CM for 12 h. The intracellular lipid content of mesangial cells was also evaluated by Oil Red O staining as described above.

#### 2.4.3. Determination of Cellular Cholesterol and Triacylglycerol Contents in Mesangial Cells

Mesangial SV40-Mes13 cells (3 × 10^5^ cells/well) were seeded in 6-well plates for 24 hours and then growth-arrested for another 24 h in FBS-free medium. Cells were treated with OA/BSA (200 *μ*M) alone or in combination with different concentrations of OF and CM for 12 h. Whole cell lysates were collected. The contents of TC and TG in mesangial cells were also measured as described above.

#### 2.4.4. Measurement of Protein Levels of TGF-*β* and Fibronectin

Mesangial cells were seeded at 3 × 10^4^ cells/well in 24-well plates with DMEM/F12 medium with 5% FBS. Subconfluent mesangial cells were made quiescent by serum deprivation for 24 h before treatment. Cells were then treated with OA/BSA (200 *μ*M) alone or in combination with different concentrations of OF or CM for 6 h incubation (for the determination of TGF-*β*) and for 24 h incubation (for the determination of fibronectin). Cell-free supernatants were collected, and the concentrations of TGF-*β* and fibronectin in supernatants were quantified using the commercial TGF-*β* ELISA kit (Bender MedSystems GmbH, Vienna, Austria) and the fibronectin ELISA kit (Assaypro, Winfield, MO, USA), respectively, following the protocols from the manufacturers.

#### 2.4.5. Immunofluorescence Staining

Mesangial SV40-Mes13 cells (2 × 10^4^ cells/well) were seeded onto 8-well Lab-Tek II chamber slides (NUNC, Rochester, NY). After serum starvation, cells were treated with OA/BSA (200 *μ*M) alone or in combination with 200 *μ*g/mL of OF or CM. After 24 h incubation, mesangial cells were washed with cold PBS, fixed with acetone/methanol (1/1; *v*/*v*), and then stained. After 1 h blocking with 3% BSA in PBS with 0.1% Triton X-100 (PBST), the cells were incubated with a primary antibody (rabbit anti-mouse fibronectin antibody, Epitomics, CA, Burlingame, USA) overnight, washed with PBS, and incubated with the DyLight 488-conjugated secondary antibody (Jackson ImmunoResearch, West Grove, PA, USA) for 2 h. The slides were mounted with 87% glycerol and imaged using a DeltaVision® Core live-cell microscope (Applied Precision Inc., WA, USA).

#### 2.4.6. Quantitative RT-PCR Analysis of Fibronectin mRNA Level

Mesangial SV40-Mes13 cells (1 × 10^6^ cells/well) were seeded in a 6 cm dish for 24 h incubation. After serum starvation, cells were treated with OA/BSA (200 *μ*M) alone or in combination with various concentrations of OF or CM. After 24 h incubation, cells were lysed in the TRIzol reagent (Invitrogen) and total RNA was isolated. Then, RNA was analyzed by RT-PCR as previously described. The upstream and downstream PCR primers for fibronectin were designed as 5′-GCT TCA TGC CGC TAG ATG T-3′ and 5′-GTG TGG ATT GAC CTT GGT AGA G-3′, respectively. In this experiment, the gene of *β*-actin was selected as a reference. The sequences of the upstream and downstream PCR primers for *β*-actin were 5′-GGA CTC CTA TGT GGG TGA CG-3′ and 5′-CTT CTC CAT GTC GTC CCA GT-3′, respectively. These primer pairs amplified a 99 bp fragment of the fibronectin cDNA and a 102 bp fragment of the *β*-actin cDNA, respectively. The fibronectin mRNA level was normalized to that of *β*-actin mRNA. Fold expression was defined as the fold increase relative to control cells.

### 2.5. Statistical Analysis

All data are presented as the mean ± standard deviation (SD). Statistical analyses were performed using the SPSS 23.0 statistical package (Chicago, IL, USA). Student's *t*-test or one-way ANOVA and Duncan's multiple comparison test were used to compare between-group differences. A *p* value of <0.05 was considered statistically significant.

## 3. Results

### 3.1. Inhibitory Effect of OF and CM on Lipid Accumulation in FFA/BSA-Treated HepG2 Cells

**Figure 1:**
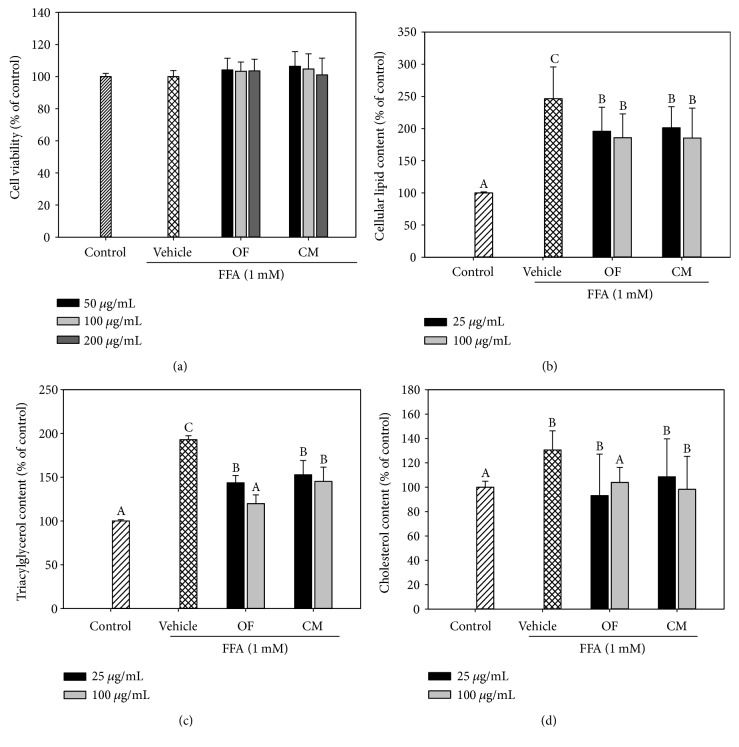
Effects of OF and CM on lipid accumulation in free fatty acid-overloaded HepG2 cells. HepG2 cells were incubated with 1 mM FFAs/BSA and cotreated with various concentrations of OF and CM for 24 h. Vehicle cells were incubated with 0.1% DMSO in the presence of FFAs/BSA. Control cells were incubated with 1% BSA. Cell viability was measured by the MTT assay (a). Quantitative analysis of lipid deposition in cells by the OD_500 nm_ values using Oil Red O staining (b). Intracellular triglyceride (c) and cholesterol (d) contents were determined in cell lysates by an enzymatic colorimetric method using a commercially available kit. Total cholesterol and TG levels of the control cells were 25.3 ± 7.1 and 28.7 ± 2.7 *μ*g/mg of cellular protein, respectively. Data were presented as mean ± SD of three independent experiments. Values not sharing common superscripts are significantly different (*p* < 0.05).

A cellular hepatic model to study hepatic steatosis *in vitro* has been established by treating human HepG2 cells with FFAs. Herein, we used a mixture of different proportions of saturated and unsaturated FFAs as described by Lin et al. [17] to examine the antilipotoxic effect of OF and CM. To determine whether the treatment of OF or CM on HepG2 cells has an apparent toxic effect, the cell viability of HepG2 cells treated with various concentrations of OF or CM in the presence of FFAs/BSA (1 mM) was determined. The results showed that after 24 h, treatment with OF or CM at concentrations up to 200 *μ*g/mL did not significantly cause cell death with respect to control cells (Figure 1(a)). Hence, the concentrations of 25 and 100 *μ*g/mL for OF and CM were used in subsequent experiments.

As shown in Figure 1(b), OF and CM attenuated FFA/BSA-induced intracellular lipid deposition in HepG2 cells. Next, we investigated whether both extracts inhibited intracellular accumulation of TG and cholesterol. OF and CM treatment significantly reduced FFA/BSA-induced cellular triacylglycerol accumulation (Figure 1(c)). OF treatment (100 *μ*g/mL) significantly reduced cholesterol content, while the reductions by CM treatment did not reach statistical significance (Figure 1(d)).

**Figure 2:**
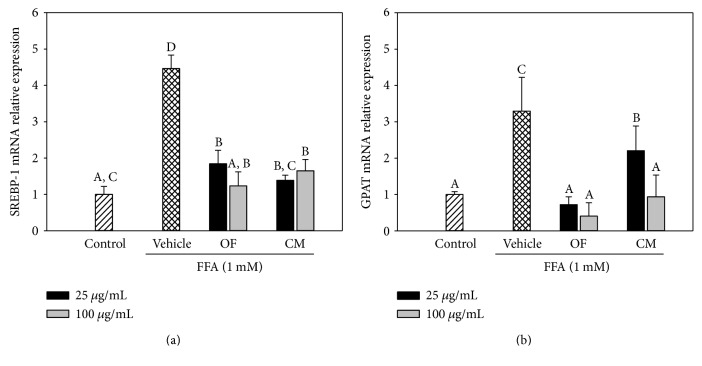
Effects of OF and CM on the mRNA expressions of lipogenesis-related genes. Real-time RT-PCR analysis of sterol regulatory element-binding protein-1 (*SREBP-1c*) (a) and glycerol-3-phosphate acyltransferase (*GPAT*) (b) mRNA levels in 1 mM FFA/BSA-treated HepG2 cells. All data were normalized to *GAPDH* mRNA, and the fold changes in expression were calculated relative to control cells (treated with 1% BSA). Each experiment was independently performed three times. Data were presented as the mean ± SD. Values not sharing common superscripts are significantly different (*p* < 0.05).

Since both extracts possessed a significantly inhibitory effect of lipid accumulation, we tried to find out the molecular target(s) of OF and CM in the lipogenesis-related genes. As shown in Figure 2, treatment of OF or CM significantly inhibited the mRNA expression of SREBP-1c and GPAT as compared to the vehicle-alone treatment.

### 3.2. Inhibitory Effect of OF and CM on FFA/BSA-Induced ROS Production in HepG2 Cells

**Figure 3:**
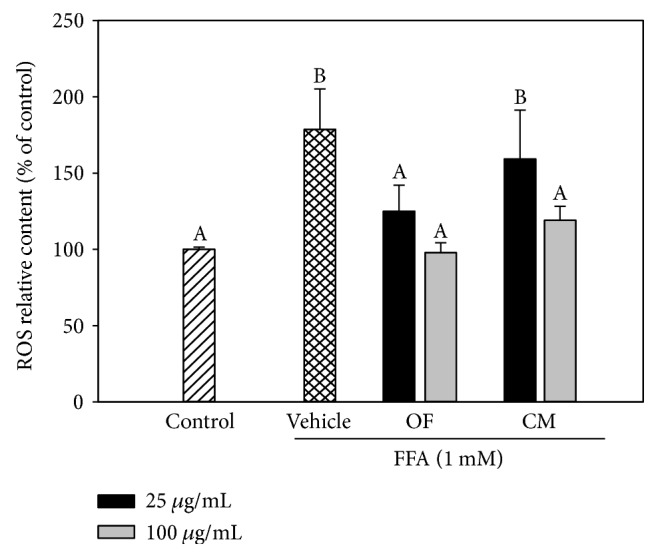
Effect of OF and CM on FFA-induced ROS production. HepG2 cells were incubated with 1 mM FFAs/BSA for 24 h in the presence of OF or CM. Vehicle cells were incubated with 0.1% DMSO in the presence of FFAs/BSA. Control cells were incubated with 1% BSA. Intracellular ROS production was quantified using the fluorescent probe DCFDA. Data were presented as mean ± SD of three independent experiments. Values not sharing common superscripts are significantly different (*p* < 0.05).

Excess ROS accumulation plays a causative role in a variety of lipotoxic disorders. After 24 h of treatment, vehicle cells exhibited a significant increase in ROS accumulation as measured by DCF fluorescence, while coincubation of HepG2 cells with OF or CM (25 and 100 *μ*g/mL) resulted in a significant decrease in comparison with vehicle cells, suggesting that the antilipotoxicity effect of both extracts was also related to their antioxidant properties (Figure 3).

### 3.3. Inhibitory Effect of OF and CM on Conditioned Medium-Induced Proinflammatory Cytokine Expression in HepG2 Cells

**Figure 4:**
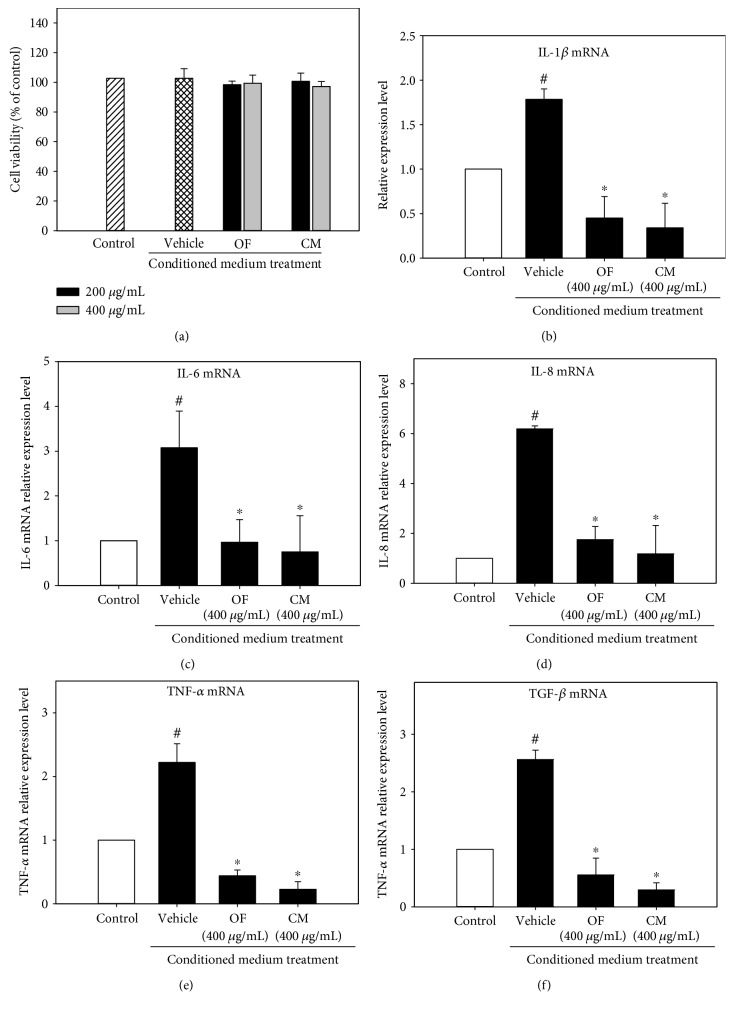
OF and CM suppressed proinflammatory cytokine mRNA expression induced by conditioned medium derived from LPS-stimulated THP-1 cells (TLPS/CM). Cell viability was measured by the MTT assay (a). Real-time RT-PCR analysis of mRNA levels of IL-1*β* (b), IL-6 (c), IL-8 (d), TNF-*α* (e), and TGF-*β* (f) in HepG2 cells cultured with RPMI medium (control cells) and 50% TLPS/CM (vehicle cells) or coincubated with 400 *μ*g/mL of OF or CM in 50% TLPS/CM. All data were normalized to GAPDH mRNA, and the fold changes in expression were calculated relative to control cells. Each experiment was independently performed three times. Data were presented as the mean ± SD. ^#^*p* < 0.05 versus control cells; ^∗^*p* < 0.05 versus vehicle cells.

Besides accumulation of triglycerides in hepatocytes, chronic hepatic inflammation is also closely associated with the pathogenesis of NAFLD. Since activated monocytes/macrophages release a wide range of proinflammatory mediators leading to induction of inflammation. In this study, the conditioned medium derived from LPS-stimulated THP-1 monocytes (TLPS/CM) was used to induce inflammatory responses in HepG2 cells. Treatment with TLPS/CM and cotreatment of TLPS/CM with OF or CM (200 and 400 *μ*g/mL) did not significantly affect cell viability with respect to control cells (Figure 4(a)). So, the concentration of 400 *μ*g/mL was used for the subsequent experiments to examine the anti-inflammatory activity of both extracts. To determine the effect of OF and CM on the mRNA expression of proinflammatory cytokines, HepG2 cells were treated with both extracts in the presence of TLPS/CM. As shown in Figures 4(b), 4(c), 4(d), 4(e), and 4(f), the mRNA expression of genes encoding proinflammatory cytokines TNF-*α*, IL-6, IL-8, IL-1*β*, and TGF-*β* was significantly increased after TLPS/CM treatment. OF or CM treatment decreased the mRNA expression of these inflammatory cytokines.

### 3.4. OF and CM Reduced Cholesterol and Triacylglycerol Contents in OA/BSA-Treated Mesangial Cells

**Figure 5:**
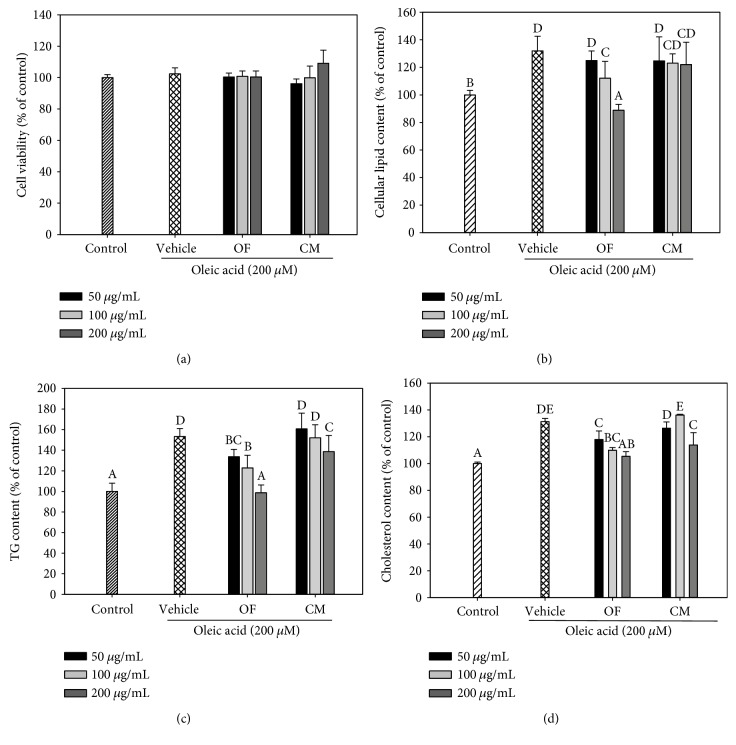
Effects of OF and CM on lipid deposit in oleic acid- (OA-) stimulated mesangial SV40-Mes13 cells. Mesangial cells were incubated with 200 *μ*M OA/BSA and cotreated with various concentrations of OF and CM for 12 h. Vehicle cells were incubated with 0.1% DMSO in the presence of OA/BSA. Control cells were incubated with 1% BSA. Cell viability was measured by the Alamar blue assay (a). Quantitative analysis of lipid deposition in cells by the OD_500 nm_ values using Oil Red O staining (b). Intracellular triglyceride (c) and cholesterol (d) contents were determined in cell lysates by an enzymatic colorimetric method using a commercially available kit. Total cholesterol and TG contents of the control cells were 9.8 ± 0.1 and 7.5 ± 0.2 *μ*g/mg cellular protein, respectively. Data were presented as mean ± SD of three independent experiments. Values not sharing common superscripts are significantly different (*p* < 0.05).

The cytotoxicity of OF and CM on mesangial cells was assessed, indicating that both extracts at the concentrations of 50, 100, or 200 *μ*g/mL did not affect cell viability (Figure 5(a)). Therefore, these concentrations of both extracts were used for further studies. As shown in Figure 5(b), OF and CM at the concentrations of 100 and 200 *μ*g/mL significantly attenuated OA/BSA-induced intracellular lipid deposition in mesangial cells. In addition, OF (50, 100, and 200 *μ*g/mL) markedly reduced TG and cholesterol contents, while CM also showed a significantly suppressive effect on TG and cholesterol accumulation but only at 200 *μ*g/mL and to a less extent (Figures 5(c) and 5(d)).

### 3.5. Effect of OF and CM on TGF-*β* and Fibronectin Levels in OA/BSA-Treated Mesangial Cells

**Figure 6:**
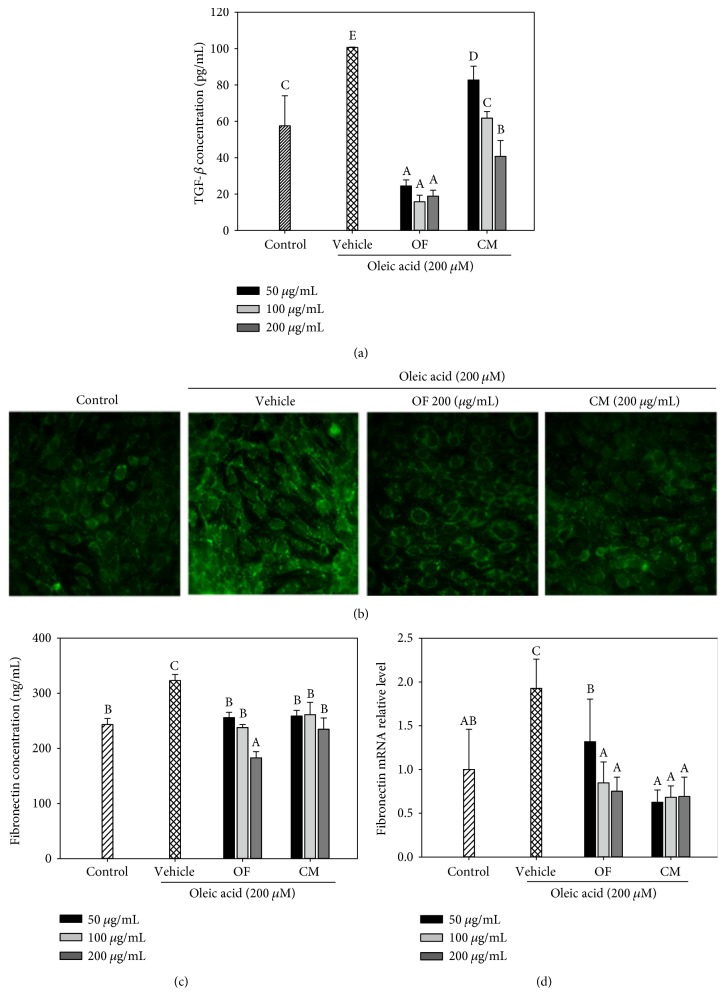
Effects of OF and CM on TGF-*β* and fibronectin expression in oleic acid- (OA-) stimulated mesangial SV40-Mes13 cells. TGF-*β* protein level in cell supernatant was measured by the ELISA method (a). Fibronectin deposition was observed by immunofluorescence staining (b). OF and CM suppressed OA-induced fibronectin protein level (c) and mRNA expression (d). Data were presented as mean ± SD of three independent experiments. Values not sharing common superscripts are significantly different (*p* < 0.05).

Given the known importance of TGF-*β* in the induction of matrix production by mesangial cells in glomerular sclerosis, we examined the inhibitory effect of OF and CM on OA/BSA-induced TGF-*β* secretion. As shown in Figure 6(a), both extracts markedly decreased TGF-*β* levels. Immunofluorescence staining showed increased presence of fibronectin in OA/BSA-treated mesangial cells. Treatments of mesangial cells with OF or CM (200 *μ*g/mL) inhibited fibronectin levels as compared to the vehicle treatment (Figure 6(b)). Hence, the effect of both extracts on fibronectin protein level was evaluated. As shown in Figure 6(c), when mesangial cells were treated with OA/BSA, fibronectin protein level was markedly elevated in the supernatant. Consistent with the immunofluorescence staining results, both extracts also reduced the secretion of fibronectin induced by OA/BSA (Figure 6(c)). Next, we observed that cotreatment with OF or CM resulted in a significant reduction in fibronectin mRNA level in comparison to the treatment with OA alone (Figure 6(d)). These data suggested that both extracts inhibited oleic acid-induced mesangial cell activation.

## 4. Discussion

Consistent with previous studies [17], treatment of 1 mM FFAs caused lipid accumulation but not cytotoxicity toward HepG2 cells (Figure 1). Lee et al. [19] reported that the methanol extract of *C. morifolium* flowers significantly inhibited lipid accumulation in 3T3-L1 adipocyte cells during differentiation. In the present study, we observed that OF and CM had an inhibitory effect on lipid accumulation in FFA-treated HepG2 cells (Figures 1(b), 1(c), and 1(d)). We next examined whether OF and CM could influence lipid metabolism through the transcriptional regulation of SREBP-1c and GPAT. SREBP-1c is a lipogenic transcription factor which upregulates acetyl-CoA carboxylase (ACC) and fatty acid synthase (FAS), which catalyze de novo fatty acid synthesis contributing to hepatic steatosis [20]. GPAT catalyzes the first and rate-limiting step in glycerolipid synthesis. It contributes to TG biosynthesis and lipid droplet formation [21]. Both OF and CM downregulated SREBP-1c and GPAT gene expression in FFA-overloaded HepG2 cells (Figure 2). Cui et al. [14] reported that polyphenol-rich *C. morifolium* extract prevented fatty liver by decreasing the expression of SREBP-1c and its target gene FAS in high-fat/propylthiouracil-fed mice. Thus, the results suggested that the lipid-lowering effect of OF and CM might be partially mediated by the downregulation of SREBP-1c and GPAT gene expression. Notably, although OF or CM treatment resulted in a significant downregulation of SREBP-1c at the transcriptional level (Figure 2(a)), further study will be still required to investigate whether both extracts regulate proteolytic processing of the inactive endoplasmic reticulum membrane-bound SREBP-1c precursor to yield its transcriptionally active N-terminal form.

Crude methanol or ethanol extract of *O. fragrans* flowers exerted antioxidant activity *in vitro* [7, 22]. The 75% ethanolic extract of *O. fragrans* flowers showed antioxidant activity by the reduction of hepatic lipid peroxidation in acetaminophen-fed mice [11]. The total flavonoids of *C. morifolium* reversed lipid peroxidation and protected the liver and kidney against lead-induced oxidative damage in mice [23]. Consistent with these previous studies, OF and CM possessed antioxidant capacity to reduce FFA-induced ROS production (Figure 3).

The intestinal microorganisms play a critical role in normal gut function and health maintenance, and the dietary composition can affect the nature of microbial colonization [2]. Evidences indicate that a high-fat diet affects gut microbiota composition accompanied with elevating plasma endotoxin levels. These alterations have been associated with hepatic steatosis and obesity [24–26]. LPS in the circulation (endotoxemia) can activate NF-*κ*B and then trigger proinflammatory signaling pathways. THP-1 cells respond with a similar transcriptional pattern as peripheral blood mononuclear cell- (PBMC-) derived macrophages after stimulation with LPS [27]. After LPS stimulation, THP-1 cells secrete increased amounts of TNF-*α*, IL-1*β*, IL-6, IL-8, and IL-10 [28, 29]. Therefore, we used a TLPS/CM-treated HepG2 cell model to mimic endotoxemia-mediated inflammation in this study. Our results showed that OF or CM treatment effectively inhibited TLPS/CM-induced mRNA expressions of proinflammatory cytokines, such as IL-1*β*, IL-6, IL-8, TNF-*α*, and TGF-*β* in HepG2 cells (Figure 4). We previously reported that water extracts of *O. fragrans* and *C. morifolium* also possessed anti-inflammatory capacity to attenuate LPS-induced nitric oxide (NO) production by RAW 264.7 macrophages [30]. Ethanol extract of *O. fragrans* flowers has been shown to obviously reduce the expression levels of the proinflammatory mediators IL-6, IL-8, and NO in LPS-stimulated human periodontal ligament cells [31]. The *n*-hexane soluble form and the nonsaponifiable lipid fractions of *C. morifolium* flower extract and its components showed marked anti-inflammatory activity against 12-O-tetradecanoylphorbol-13-acetate- (TPA-) induced ear edema in mice [32]. Taken together, OF and CM could have hepatoprotective activity by modulating fat deposition in hepatocytes and regulating the inflammatory responses to decrease the progression of steatohepatitis.

Notably, OF and CM treatments significantly suppressed TGF-*β* level induction by TLPS/CM. Since TGF-*β* inhibitors may be nephroprotective [33], we next examined the possible nephroprotective properties of OF and CM in oleate-treated mesangial cells. The prevalence of obesity-related glomerulopathy is increasing, that includes increases in mesangial matrix, thickening of the glomerular basement membrane, and glomerulosclerosis. The changes may be independent on high blood pressure and glucose, or may precede the emergence of them, and may be attributed to lipid accumulation in mesangial cells [34]. OF and CM also showed a lipid-lowering effect on OA/BSA-treated renal glomerular mesangial cells (Figure 5). Thus, the TG-lowering effect of OF and CM might contribute to the nephroprotective potential.

Mishra and Simonson [18] demonstrated that oleate raises secretions of TGF-*β*, collagen I, and fibronectin and can induce a myofibroblast phenotype in mesangial cells. Consistent with their findings, 200 *μ*M OA/BSA treatment significantly elevated TGF-*β* and fibronectin protein levels (Figure 6). Hung et al. [35] reported that the water extract of *O. fragrans* attenuated TGF-*β*1-induced intercellular/extracellular original fibronectin in human lung fibroblast cells and exerted antifibrotic activity against lung fibrosis. The hot-water extract of *C. morifolium* flowers was considered to be beneficial for type 2 diabetes [36]. In this study, we demonstrated that OF and CM can reverse the increased expression of TGF-*β* and the increased deposition and secretion of fibronectin in high-OA-treated mesangial cells (Figure 6). Taken together, OF and CM may prove beneficial in the development of natural agents for the prevention or treatment for TGF-*β*-mediated fibrosis disorders. Besides TGF-*β*, monocyte chemoattractant protein-1 (MCP-1) also contributes to ECM accumulation in the pathogenesis of diabetic nephropathy (DN) [37]. Our preliminary data showed that CM treatment (100, 200, and 400 *μ*g/mL) significantly abrogated high-glucose-induced MCP-1 protein level in mesangial cells (data not shown). Thus, further investigation of the potential protective role of OF and CM in DN will be studied in the future.

In conclusion, both flower extracts of *O. fragrans* and *C. morifolium* ameliorated FFA-induced lipid deposit and ROS and possessed anti-inflammatory activity in HepG2 cells. In addition, both extracts inhibited lipid accumulation, induction of TGF-*β*, and extracellular matrix accumulation in OA-overloaded mouse mesangial cells. These results demonstrate that both flower extracts of *O. fragrans* and *C. morifolium* may have a protective effect on nonalcoholic steatohepatitis and renal fibrosis.

## Figures and Tables

**Table 1:** List of primer pairs used for quantitative PCR.

Primer	Sequence (5′ to 3′)	Product length (bp)	Binding site
*SREBP-1* forward	CGG AGA AGC TGC CTA TCA AC	379	Exon 5
*SREBP-1* reverse	GGT CAG TGT GTC CTC CAC CT		Exon 7
*GPAT* forward	AGT GAG GAA TGG GGT GAG TG	300	Exons 3 and 4
*GPAT* reverse	CAG TCA CAT TGG TGG CAA AC		Exon 6
*IL-1β* forward	CAC ATG GGA TAA CGA GGC TT	147	Exon 5
*IL-1β* reverse	TTG TTG CTC CAT ATC CTG TCC		Exon 5
*IL-6* forward	CTC AGC CCT GAG AAA GGA GA	310	Exons 2 and 3
*IL-6* reverse	CAG GGG TGG TTA TTG CAT TCT		Exon 5
*IL-8* forward	GTG CAG TTT TGC CAA GGA GT	195	Exon 2
*IL-8* reverse	CTC TGC ACC CAG TTT TCC TT		Exon 3
*TNF-α* forward	CAC TAA GAA TTC AAA CTG GGG C	165	Exon 4
*TNF-α* reverse	GAG GAA GGC CTA AGG TCC AC		Exon 4
*TGF-β* forward	GGG ACT ATC CAC CTG CAA GA	420	Exon 1
*TGF-β* reverse	CAC GTG CTG CTC CAC TTT TA		Exon 2
*GAPDH* forward	AAA GGA TCC ACT GGC GTC TTC ACC ACC	206	Exon 5
*GAPDH* reverse	GAA TTC GTC ATG GAT GAC CTT GGC CAG		Exon 7
